# Silencing of the olfactory co-receptor gene in *Dendroctonus armandi* leads to EAG response declining to major host volatiles

**DOI:** 10.1038/srep23136

**Published:** 2016-03-16

**Authors:** Ranran Zhang, Guanqun Gao, Hui Chen

**Affiliations:** 1 College of Forestry, Northwest A&F University, Yangling, Shaanxi 712100, China

## Abstract

In this study, a polymerase chain reaction (PCR) based on homology genes of Orco was utilized to identify DarmOrco, which is essential for olfaction in *D*. *armandi*. The results showed that DarmOrco shares significant sequence homology with Orco proteins had known in other insects. Quantitative real-time PCR (qRT-PCR) analysis suggested that DarmOrco was abundantly expressed in adult *D*. *armandi*; by contrast, DarmOrco showed trace amounts of expression level in other stages. Of different tissues, DarmOrco expression level was the highest in the antennae. In order to understand the functional significance of Orco, we injected siRNA of DarmOrco into the conjunctivum between the second and third abdominal segments, and evaluated its expression after siRNA injected for 24 h, 48 h and 72 h. The results of qRT-PCR demonstrated that the reduction of mRNA expression level was significant (~80%) in DarmOrco siRNA-treated *D*. *armandi* than in water-injected and non-injected controls. The electroantennogram responses of females and males to 11 major volatiles of its host, were also reduced (30~68% for females; 16~70% for males) in siRNA-treated *D*. *armandi* compared with the controls. These results suggest that DarmOrco is crucial in mediating odorant perception.

Olfaction is one of the most important sensory modalities in insects. Behaviors such as looking for food, mating, choosing oviposition sites, escaping from predators and so on depend almost exclusively on the correct distinguishing of chemical signals from the environment. The olfactory systems of insects, completely differs from those of vertebrates^1^. Insect ORs can act as ionotropic receptors when a conventional OR combines with an odorant receptor co-receptor to form a ligand-gated cation channel^2^,^3^,^4^,^5^. Orco, which is short for “olfactory receptor coreceptor”^6^, is known as OR83b in *Drosophila melanogaster*, OR2 in *Bombyx mori*, and OR7 in mosquitoes in the past. A functional OR is a heterodimer formed by a specific OR and a ubiquitous co-receptor named odorant receptor co-receptor or Orco^6^,^7^. Orco has been described in insects belong to different orders, as a unique gene that is characterized by an extremely highly conserved seven transmembrane domain (7TM) based on amino acid sequence; specifically, the co-receptor shares a sequence identity up to 94% with orthologs in other insects^8^,^9^,^10^,^11^,^12^,^13^. This high identity suggests that Orco exhibits similar functions across different insect taxa. In fact, Orco is suggested to be responsible for the OR adopting the correct structure, and it also works as a selective ion channel during olfactory signal transduction^14^,^15^,^16^. Recent studies have demonstrated that any disturbance in Orco expression induces a complete disruption in the insect olfactory system^17^,^18^,^19^,^20^.

RNAi is a powerful tool that is used to understand various aspects of insect physiology^7^,^21^,^22^,^23^. By silencing different genes, the roles of diverse proteins in the olfactory signaling pathways have been unraveled^18^,^19^,^24^,^25^,^26^.

The Chinese white pine beetle, *Dendroctonus armandi* (Tsai and Li), is an important forest pest in China, which not only intrude into health *Pinus armandi* Franch aged 30 years or more but also help other beetles to attack the host trees, causing serious loss of *P*. *armandi*^27^,^28^. *D*. *armandi*, like most insects, locate their hosts mainly depend on olfactory signals. It is clear that various of beetles in *Dendroctonus* use both attractants and antiattractants, that emanate from host and non-host plants, as well as from conspecific and heterospecific bark beetle individuals, to accomplish their mission of mass attacks^29^,^30^,^31^,^32^. However, the attractants and antiattractants of *D*. *armandi* are still unknown. It is exciting that a recent research of Guofa Chen^33^ showed that: (1) *D*. *armandi* females can produce four potential pheromone compounds, (−)-trans-verbenol (tV; major component), exo-brevicomin (EBV), seudenol (SD), and 1-methyl-2-cy-clohexen-1-ol (MCOL); (2) The four potential pheromone compounds and five major host monoterpenes and one sesquiterpene (α-pinene, (+)-camphene, β-myrcene, D-Limonene, (+)-Longifolene) were noneffective in trap experiment when used alone but when they were mixed together the effectiveness increased significantly (4–10 times) However, the mechanism by which this chemical signaling works in this species remains unknown. Due to the high degree of conservation of the Orco, we were able to identify a unique putative DarmOrco gene. Furthermore, the expression profiles of DarmOrco were evaluated in different development stages and different tissues of *D*. *armandi* by using qRT-PCR. We examined the efficiency of Orco silencing by analyzing the expression of DarmOrco mRNA. To investigate the function of the DarmOrco gene in *D*. *armandi* we also investigated whether the siRNA injected treatment affects electrophysiological responses of *D*. *armandi* to major volatiles of its host.

## Results

### DarmOrco sequence and topology analysis

**Figure 1:**
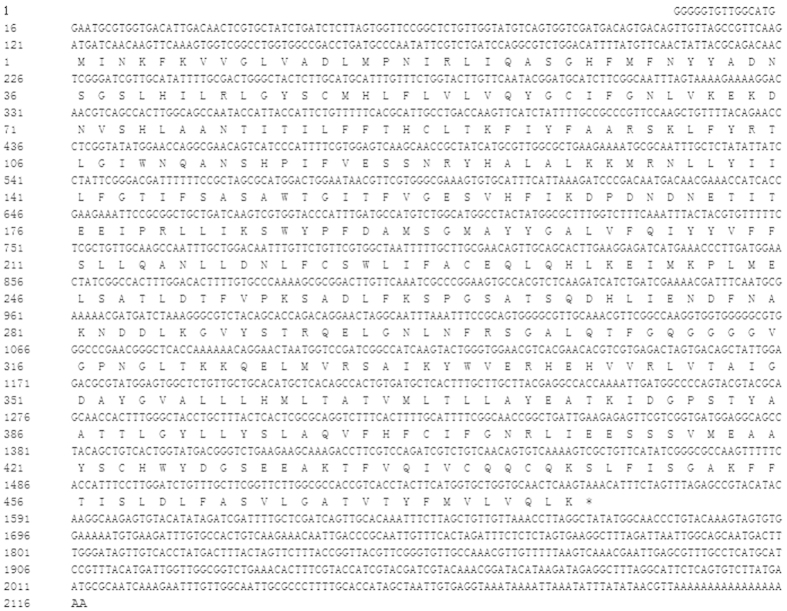
Nucleotide sequence and putative amino acid sequence of the *D*. *armandi* Orco.

**Figure 2:**
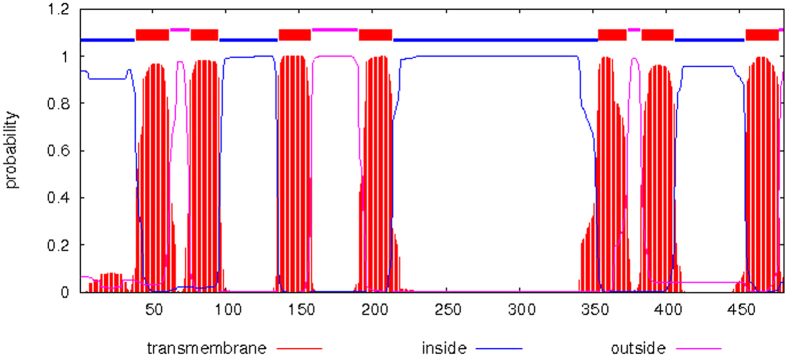
Predicted transmembrane topology of DarmOrco. The transmembrane region is in red; the inside is in blue; the outside is in purple.

We successfully obtained an Orco cDNA fragment by PCR using degenerate primers. RACE PCR was used to amplify the remaining 5′- and 3′-ends of the Orco gene. In order to generate the full-length Orco gene named as DarmOrco, the sequence obtained from 5′- and 3′- RACE PCR was assembled with the original Orco fragment. The full length of the DarmOrco gene is 2117 bp with an open reading frame (ORF) of 1443 bp. The 5′ untranslated region (UTR) and the 3′ UTR are 120 bp and 554 bp, respectively (Fig. 1). The DarmOrco ORF encodes a protein of 480 amino acids that has high sequence identity with Orco proteins from various insect species across a 4 different orders. The DarmOrco protein shares a 97% amino acid identity with the Orco of *D*. *ponderosae* (JQ855701.1), 84% amino acid identity with *T. castaneum* Orco (XP-008194693.1), 83% amino acid identity with *T*. *molitor* Orco (AJQ66219.1) and *A*. *quadriimpressum* (AJF94638.1). The signal peptide, molecular weight, isoelectric point and phosphorylation sites of the DarmOrco were predicted. The ORF encoded 480 amino acids, lacking a signal peptide but have 10 phosphorylation sites (Ser160, Ser185, Ser263, Ser266, Ser382, Ser415 and Ser422; Thr321; Tyr32 and Tyr125). The molecular weight of the peptides was 54.03 kD and the isoelectric point (pI) was 6.94. Regarding amino acid composition, 37 positively charged residues (Arg + Lys) and 38 negatively charged residues (Asp + Glu) were present. The instability index (II) is computed to be 34.09 which classifies the protein as stable. The Aliphatic index and the grand average of hydropathicity (GRAVY) were 99.98 and 0.258, respectively indicating that the polypeptides had a high hydrophobicity and this is consistent with the characteristics of membrane proteins. The membrane topology analysis of the DarmOrco protein predicted by TMHMM2.0 indicated that this protein is a seven transmembrane protein with an intracellular N-terminus and an extracellular C-terminus (Fig. 2), which is consistent with the membrane topology of Orco protein had demonstrated in both *D. melanogaster* and *A. gambiae*^34^.

**Figure 3:**
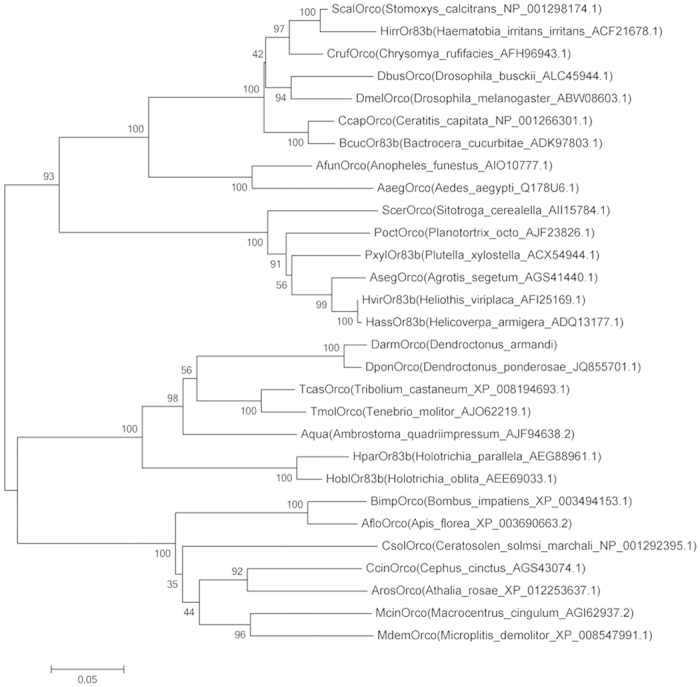
Phylogenetic tree of Orco orthologs from various insect species. The tree was constructed withMEGA5.05 using the neighbor-joining (NJ) method in MEGA 5.05. Values indicated at the nodes are bootstrap values based on 1000 replicates.

**Figure 4:**
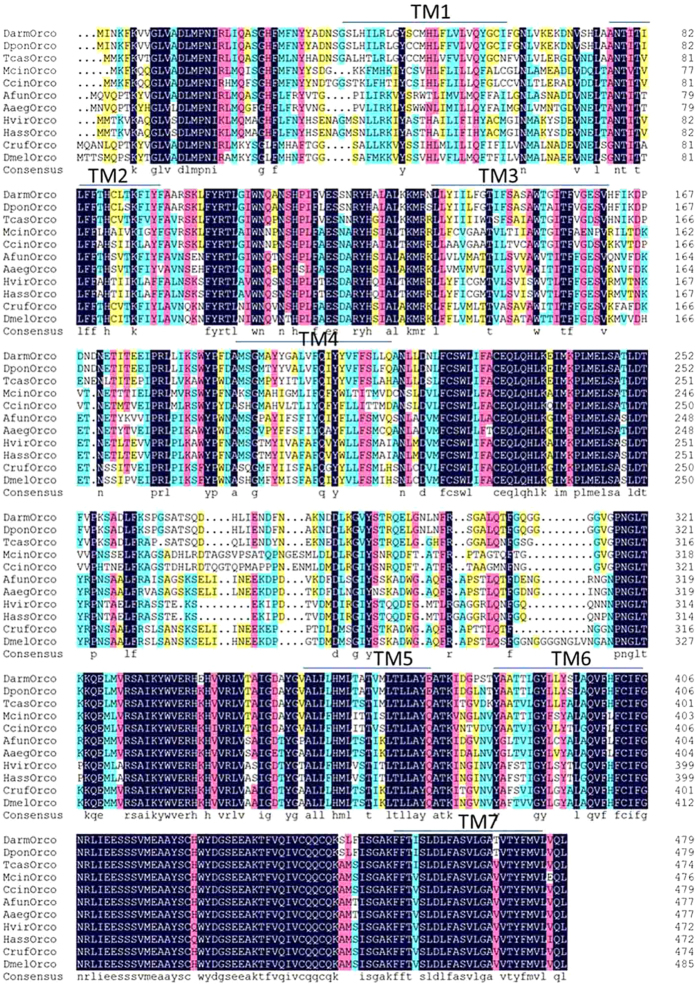
Amino acid sequence alignment of DarmOrco with representative Orco orthologs. Abbreviations (accession number in parentheses): Darm, *Dendroctonus armandi*; Dpon, *Dendroctonus ponderosae* (AFI45064.1-Coleoptera); Tcas, *Tribolium castaneum* (XP_008194693.1-Coleoptera); Dmel, *Drosophila melanogaster* (ABW08603.1-Diptera); Cruf, *Chrysomya rufifacies*, (AFH96943.1- Diptera); Mcin, *Macrocentrus cingulum* (AGI62937.2-Hymenoptera); Ccin, *Cephus cinctus* (AGS43074.1-Hymenoptera); Afun, *Anopheles funestus* (AIO10777.1-Diptera); Aaeg, *Aedes aegypti*, (Q178U6.1-Diptera); Hvir, *Heliothis viriplaca*, (AFI25169.1-Lepidoptera); Hass, *Helicoverpa armigera*, (ADQ13177.1- Lepidoptera).

A phylogenetic tree was constructed by using 29 insect Orco protein sequences. The protein accession numbers are given in Fig. 3. The phylogenetic tree was divided into two big branches, one coved Coleoptera and Hymenoptera, the other coved Lepidoptera and Diptera. As expected, *D*. *armandi* Orco was rooted in the Coleoptera group with *D*. *ponderosae*, *T*. *castaneum*, *T*. *molitor* and *A*. *quadriimpressum*. Sequence similarities between DarmOrco and Orco from different insect orders (Coleoptera, Lepidoptera, Hymenoptera and Diptera) reached a relatively high amino acid identity (97%, 63%, 67%, and 67%, respectively) with each other (Fig. 4). This result suggested Orco is important in insect odorant perception. In addition, residues that comprised C-terminus (portions of trans-membrane helices (TMs 6 and 7) were reasonably conserved with Orco of other insects in terms of position and side-chain character. We compared DarmOrco with other Orco protein sequences from insects of different orders and the results showed a very high level of conservation and a relationship among the Orco subtypes within insect orders (Fig. 3).

### Tissue and developmental expression of DarmOrco transcript

**Figure 5:**
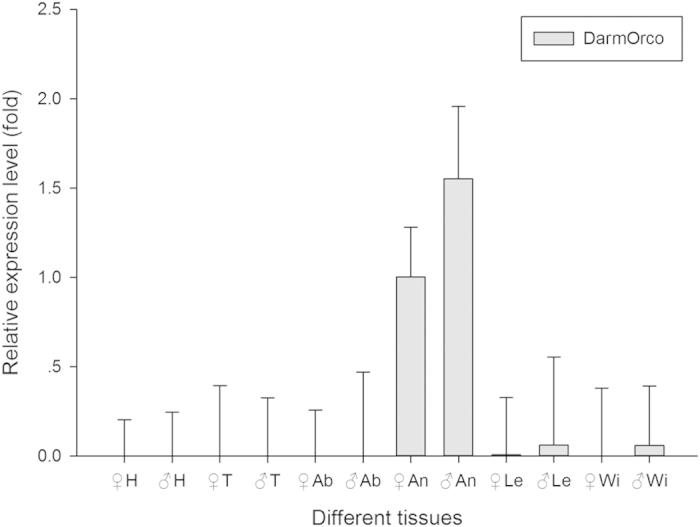
Relative expression levels of DarmOrco in different tissues. The relative expression levels were normalized by actin, with the expression of female antennae as the calibrator. The standard errors of the means of three biological replicates are represented by error bars. ♀H, female heads; ♂H, male heads; ♀T, female thoraces; ♂T, male thoraces; ♀An, female antennae; ♂An, male antennae; ♀Ab, female abdomen; ♂Ab, male abdomen; ♀Le, female legs; ♂Le, male legs; ♀Wi, female wings; ♂Wi, male wings.

**Figure 6:**
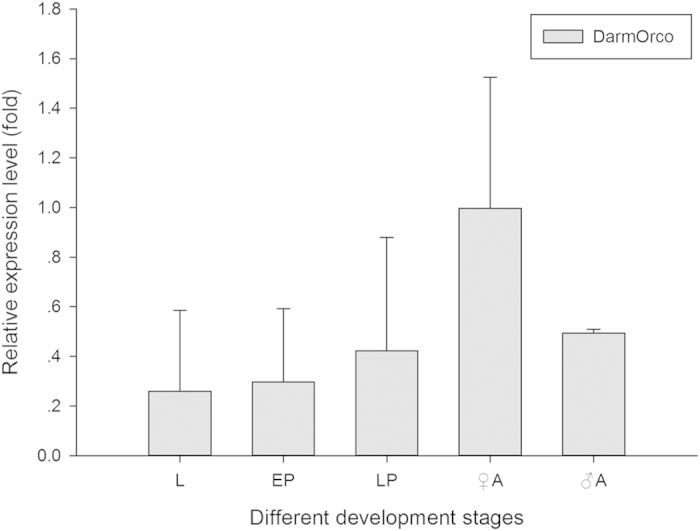
Relative expression levels of DarmOrco in different developmental stages. The relative expression levels were normalized by actin, with the expression of female antennae as the calibrator. The standard errors of the means of three biological replicates are represented by error bars. L, larvae; EP, early pupae; LP, later pupae; ♀A, female adult; ♂A, male adult.

We examined the expression level of the DarmOrco in different development stages and in various tissues of *D*. *armandi* adults by qRT-PCR to explore its involvement in insect olfaction along with other ORs. The DarmOrco gene was expressed mainly in the antennae of both sexes (Fig. 5). Moreover, there was no apparent difference in DarmOrco expression in male and female antennae (F = 1.836, df = 1, P = 0.247). The expression level of head, thorax and abdomen were almost zero. DarmOrco also expressed in legs and wings, although the the expression level were very low. DarmOrco was expressed in all of stages, showing the highest expression level in the adult stage (Fig. 6). Although the expression level of larval, early pupa and late pupa stage were not as more as that of adult period, but theirs expression were also very high. Compared with the female adult, the expression level of larval, early pupa and late pupa can reach 0.27-fold, 0.30-fold and 0.43-fold of female adult.

### Effect of siRNA treatment on DarmOrco transcript level

**Figure 7:**
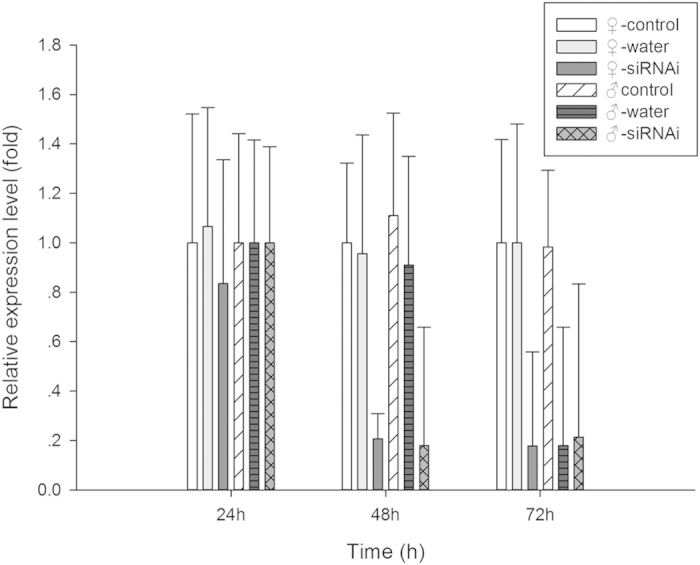
qRT-PCR analysis of DarmOrco transcript patterns from *D*. *armandi*; after injected for 24 h, 48 h and 72 h. The standard errors of the means of three biological replicates are represented by error bars.

We knocked down the DarmOrco gene by RNAi to study its function in host volatile detection. There was no difference among non-injected, water-injected and siRNA-injected groups 24 h after siRNA injection (F_♀-24 h_ = 0.401, d.f. = 2, 6, P = 0.687; F_♂-24 h_ = 0.180, d.f. = 2, 6, P = 0.839). But in 48 h and 72 h treatments , the expression level of DarmOrco was reduced by 80% compared with water-injected and non-injected controls (F_♀-48 h_ = 31.498, d.f. = 2, 6, P < 0.0001; F_♂-48 h_ = 11.416, d.f. = 2, 6, P < 0.005; F_♀-72 h_ = 29.831, d.f. = 2, 6, P < 0.0001; F_♂-72 h_ = 8.540, d.f. = 2, 6, P < 0.005) (Fig. 7). The transcript levels of DarmOrco in the non-injected or water-injected *D*. *armandi* remained unchanged.

### Effect of siRNA treatment on electrophysiological responses to semiochemicals

**Figure 8:**
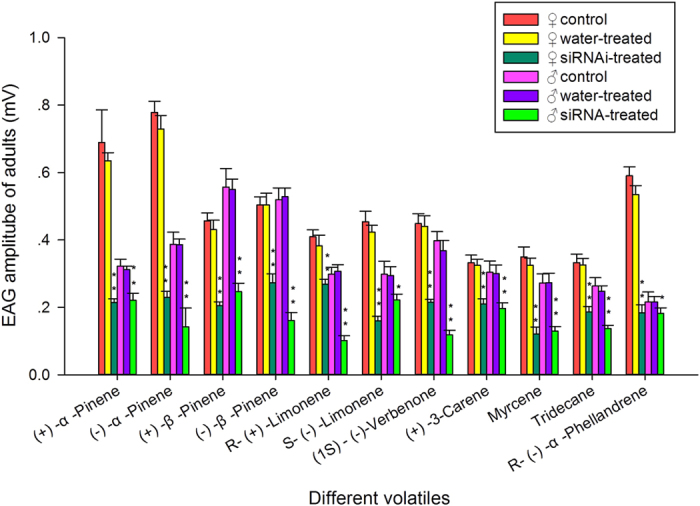
Electroantennographic (EAG) responses of siRNAi-treated water-injected, and non-injected *D*. *armandi* to 11 host volatiles. P-value, *P < 0.05; **P < 0.001.

We examined the responses of siRNA-injected, water-injected, and non-injected *D*. *armandi* to (+)-α-Pinene, (−)-α-Pinene, (+)-β-Pinene, (−)-β-Pinene, R-(+)-Limonene, S-(−)-Limonene, (1S)-(−)-verbenone, (+)-3-Carene, Myrcene, Tridecane, and R-(−)-α-Phellandren by electroantennographic analysis. The α-pinene, 3-carene, β-pinene, limonene and myrcene as the major monoterpenes were plant volatiles released by several host plants of bark beetles^35^,^36^,^37^,^38^. The response level of the siRNA-treated femals to (+)-α-Pinene, (−)-α-Pinene, (+)-β-Pinene, (−)-β-Pinene, R-(+)-Limonene, S-(−)-Limonene, (1S)-(−)-verbenone, (+)-3-Carene, Myrcene, Tridecane, and R-(−)-α-Phellandren were remarkably lower than those of the water-injected and non-injected controls with a reduction of 66.2%, 68.5%, 52.4%, 45.7%, 29.7%, 62.1%, 51.2%, 35.3%, 62.7%, 42.8% and 65.6%, respectively (P < 0.0001) (Fig. 8). Compared with females the reduction of males were 29.0%, 63.2%, 55.2%, 69.6%, 66.8%, 24.6%, 67.9%, 34.4%, 52.6%, 44.6% and 15.7% in the same turn (P_S-(−)-Limonene_ < 0.005, P_R-(−)-α-Phellandren_ < 0.005, P_others_ < 0.0001) (Fig. 8).

## Discussion

DarmOrco, the ortholog of Orco in *D*. *armandi*, was successfully cloned and characterized in this study. Despite the 7-TM domains of Orco from different insect species have evolved for millions of years, they also exhibited up to 94% homology^9^,^10^,^11^,^13^,^39^,^40^ suggesting that this protein play similar role in olfactory signal transduction in each of the different insect species. Previous research has confirmed that diverse Orco genes can functionally complement Orco-deficient *D. melanogaster*^39^. After interacting with ligand-specific ORs, Orco and ligand-specific ORs can formed heteromeric complexes, which are ligand-gated ion channels that allow odorants to pass through the cell membrane^41^,^42^,^43^.

Alignment of the DarmOrco amino acid sequence with Orco sequences from four insect orders (Coleoptera, Lepidoptera, Hymenoptera and Diptera) revealed significant sequence conservation (Fig. 4). DarmOrco is six amino acids shorter than DmelOrco and seven residues (^314^ GNGLVNG ^320^ of DmelOrco) are located in the predicted intracellular loop connecting TM4 and TM5 (Fig. 4), a region thought to be important for intracellular transport^34^. Conservation of residues within the C-terminus (predicted TM6–TM7) has been observed for conventional *Drosophila* ORs and insect Orco sequences, and Benton *et al*.^34^ demonstrated that the loop connecting TM6 and TM7 is part of a region that is thought to mediate Orco interactions with conventional ORs. The predicted TM6 and TM7 of DarmOrco display a high level of sequence conservation with other insect Orcos, including the tyrosine residue in TM7 (Y478 in *D*. *melanogaster*) that is important for successful OR-Orco interactions *in vivo*^43^. Sequence conservation within the C-terminal region of DarmOrco may translate to functional conservation, suggesting they may be able to dimerize with ligand/odorant-selective ORs. A mutation (Y464A) in TM7 of the *Bombyx mori* Orco (BmOrco) in combination with BmOr-1 results in a small increase in K^+^ selectivity^43^.

Kumar *et al*.^44^ examined several D466 substitution variants in their study, only D466E displayed significant responses to VUAA1 stimulation. The importance of this residue is further supported by the observation that D466E variant channels are more sensitive in the response to both a direct activator of Orco (VUAA1), and conventional Or-mediated ligands (methyl hexanoate and eugenol). This may be the result of the inductive effect of additional carbon in the glutamic acid R-group that gives rise to significantly higher pKa than aspartic acid, or the extra carbon could simply allow for greater flexibility that might have a role in channel gating.

Initially, the expression profiles of DarmOrco in the including antennae, heads (without antennae), thoraces, abdomens, legs, and wings were investigated at the adult stages. As shown in Fig. 5, DarmOrco is only highly expressed in the antennae of both males and females at levels that significantly differ those in other tissues (p < 0.0001). This result was not surprising, as all previous studies of Orco using quantitative PCR indicated that the gene is expressed almost exclusively in the antennae^13^,^19^,^45^. An exception is Orco (formerly known as Or7) of mosquitoes, which is expressed in gustatory tissues in addition to the antennae^10^,^11^,^12^,^46^. As observed in different insects^10^,^13^,^22^, DarmOrco is also expressed during all developmental stages (Fig. 6), from the larvae stage to adult stages, suggesting that this receptor is important during all stages of *D*. *armandi* .

The qRT-PCR results of RNAi experiment demonstrated that the level of mRNA expression in the DarmOrco siRNA-treated *D*. *armandi* was significantly reduced (70%) than that in the two controls (Fig. 7). We examined the responses of siRNA-injected, water-injected, and non-injected *D*. *armandi* to 11 host volatiles, by EAG analysis (Fig. 8). The responses of the siRNA-treated females and males to these volatiles were significantly lower than those of the controls, howere, the degree of the reductions were different in different odors or same odors in different sexes. For example, the reductions of R-(+)-Limonene were 29.7% for females but it reached 66.8% for males, meanwhile, the reductions of (+)-α-Pinene, S-(−)-Limonene and R-(−)-α-Phellandren were 66.2%, 62.1% and 65.6% for females but 29.0%, 24.6% and 15.7% for males. This result indicates that silencing DarmOrco has direct effects on female and male antennal electrophysiological responses to host volatiles. Females of *D*. *armandi* may play more important roles in distinguishing (+)-α-Pinene, S-(−)-Limonene and R-(−)-α-Phellandren. But males are possibly more sensitive to R-(+)-Limonene. Previous research has shown that females of many beetles in Scolytidae play a role in host seeking and α-Pinene and Limonene significantly (4–10 times) increases the *D*. *armandi* trap catches than captures in the blank control traps^33^, these results can explain why females reduced more than males in responses of EAG. As we know, insects can give different reactions to chiral material in the form of left-handed and right-handed, this may provide an explanation for the phenomenon males reduced more in respone to R-(+)-Limonene. In addition, significant differences in EAG responses to the chemicals were found between the siRNA-injected and non-injected treatment groups. The partial silencing of DarmOrco shown by qRT-PCR and EAG analyses demonstrated the feasibility of significantly reducing DarmOrco gene expression using siRNA.

Ideally, siRNAs would be absolutely specific, regulating only the target gene of interest. However, a growing body of evidence suggests that this is not necessarily the case^47^. These reports indicate that siRNAs can affect the expression of unintended targets. Nonetheless, the potential for off-target silencing does not override the enormous potential of RNAi as a tool for investigation of gene function^48^.

The simplest explanation for these findings is that Orco is important for the sensitivity of the insect olfactory system. Although we were not able to completely silence DarmOrco, the partial knockdown clearly affected antennal response to terpene and alkene odors. These RNAi experiments are evidence *in vivo* that DarmOrco is involved in odor reception. The results of this study may serve as a foundation for future studies that aim to target Orco orthologs to interfere with insect mate-seeking and host-locating behavior. Such non-insecticidal approaches are important in integrated pest management strategies and broaden the arsenal of available tools for insect pest control.

Insect olfactory sensory neurons (OSNs) are enclosed in sensory hairs called sensilla, which cover the surface of olfactory organs. The OSN dendrites express odorant receptor (OR) proteins, which in insects function as odor-gated ion channels^2^,^3^. The interaction of odorants with ORs either increases or decreases the basal firing rate of the OSN. This neuronal activity in the form of action potentials embodies the first representation of the quality, intensity, and temporal characteristics of the odorant^49^,^50^. We can’t explain the roles of specific ORs directly as others had done in *D*. *melanogaster*^51^,^52^,^53^,^54^ and *B*. *mori*^54^, according to our existing experimental conditions. But we can discuss the roles of various ORs of other insects and this may give us guidance in our future studies.

*B*. *mori* silkworms are attracted to mulberry leaves, Tanaka *et al*.^54^ tested the odorants responses of BmOrs and found that seven larval BmOrs (BmOr-8, 24, 29, 42, 54, 56, and 63) demonstrated current responses to at least one of the Odorants. BmOr-29 can response to linalool and citral that are structurally similar to myrcene, however, BmOr-42 response only to linalool, so we can infer that *D*. *armandi* may have some ORs homologous with BmOr-29 and 42 and it may response to linalool and citral. In *D*. *melanogaster* Or67b respond to green leaf volatiles such as (Z)-3-hexenol^55^, to which *S*. *flava* also has a robust antennal response; Or9a is activated by a broad range of ketone-, alcohol-, and carboxylic acid-containing ligands^56^. We did not test adorants of alcohol-, and carboxylic acid-containing ligands in our research but we will do it in the future and hope to find other ORs denes.

All previous research provides us with theoretical basis and guide us to work and we hope with the accumulation of knowledges and progress of the research of *D*. *armandi*, we will know more about it in the near or far future and at that we would control them in a better way.

## Conclusion

We have demonstrated the existence and characterization of an Orco gene in the *D*. *armandi*. The molecular characterization of DarmOrco and the analysis of the expression pattern is the first step to understanding the molecular mechanisms responsible for potential pest control methods. The functional characterization of the DarmOrco by RNAi demonstrates that Orco is very important in identifying the volatile semiochemicals emitted of the host. Further studies on how Orco binds these volatile chemicals and delivers this signal to neurons are needed for a complete understanding of the concerted evolution between insects and its host.

## Methods

### Insect rearing and tissue collection

1-1.5 meters high logs of infested *P*. *armandi* were collected at the Huoditang Experimental Forest Station of the Northwest A & F University. The collection site was located on the southern slope of the middle Qinling Mountains (33°18′–33°28′N, 108°21′–108°39′E), Shaanxi, China. The logs were collected in the middle of May and August, 2014 and May, 2015.

Whole bodies of *D*. *armandi* in different developmental stages (larvae, earlier pupae, later pupae and adults; 10 each) were collected, frozen in liquid nitrogen immediately, and then stored at −80 °C until use. The same procedure was done for body parts, namely, antennae, head (without antennae), legs, wings (100 for each sex), thoraxes and abdomens (10 for each sex), of adult individuals. All of the samples above were collected in triplicate.

### Total RNA isolation and cDNA synthesis

Total RNA was extracted following the protocol of the RNA extraction kit (UNlQ-10 Column Trizol Total RNA Isolation Kit, Sangon Biotech, Shanghai, China). The quality of total RNA was detected by NanoDrop ND-1000 Spectrophotometer (Nano Drop Products, Wilmington, DE, USA). Finally, 500 ng total RNA (OD260/OD280 = 1.80–2.10) was used to cDNA synthesis. First-strand cDNA was synthesized by using Prime Script^TM^ RT reagent Kit with gDNA Eraser (Perfect Real Time) (Takara Biotech, Dalian, China) according to the manufacturer’s instructions. The synthesized cDNA was stored at −80 °C until use.

### Rapid amplification of cDNA ends (RACE) to obtain full-length DarmOrco gene

Gene-specific primers were designed based on the sequence of Orco gene from *Dendroctonus ponderosae* (JQ855701.1), *Tribolium castaneum* (XP_008194693.1), Cephus cinctus (AGS43074.1), *Anopheles funestus* (AIO10777.1), *Holotrichia parallela* (AEG88961), *Holotrichia oblita* (AEE69033), *Helicoverpa armigera* (ADQ13177.1), *Drosophila busckii* (ALC45944.1) and *Bactrocera cucurbitae* (ADK97803.1) to clone the DarmOrco gene (Table 1). PCR reactions were carried out in a final mixture volume of 50 μL, containing 25 μL 2 × Taq Master Mix (CoWin Biotech, Beijing, China), 0.5 μL of each degenerate primer (10 μM, Sangon Biotech, Shanghai, China), 1 μL 1st cDNA template (synthesized using 500 ng antenna total RNA) and 23 μL RNase-free water. The PCR cycling conditions were as follows: 95 °C for 2 min, followed by 35 cycles of 94 °C for 30 s, 55 °C for 30 s, extension at 72 °C for 60 s, and a final extension at 72 °C for 10 min. PCR products were electrophoresed on a 1.0% agarose gel and visualized by GelRed staining. DNA bands of the expected length were gel-purified and cloned into the pMD18 (Simple) T-vector (Takara Biotech, Dalian, China), and the constructs containing the DarmOrco gene fragment were sequenced in both directions (TransGene, Nanjing, China).

Gene-specific primers were designed for 5′ and 3′-RACE PCR based on the obtained DarmOrco fragment (Table 1). The 5′ and 3′ regions of the target gene were amplified using a SMARTerTM RACE cDNA amplification kit (Clontech, Mountain View, CA, USA) following the manufacturer’s instructions. Touchdown PCR was performed as follows: 95 °C for 2 min; 5 cycles of 94 °C for 30 s and 72 °C for 2 min; 5 cycles of 94 °C for 30 s, 70 °C for 30 s, and 72 °C for 90 s; 30 cycles of 94 °C for 30 s, 68 °C for 30 s, and 72 °C for 90 s; and a final incubation at 72 °C for 10 min. The RACE PCR products were cloned into the pMD18 (Simple) T-vector (TaKaRa, Dalian, China) and then sequenced. Full-length DarmOrco sequence was determined by assembling the DarmOrco cDNA fragments and the sequence obtained from the 5′ and 3′-RACE PCR. Gene-specific primers encompassing the putative start and stop codons (Table 1) were designed to obtain the full length gene of DarmOrco.

### Sequence analysis and comparison

The DarmOrco gene sequence was compared with database sequences using BLASTx (http://blast.ncbi.nlm.nih.gov/). Protein sequences were aligned by using ClustalW2 (http://www.ebi.ac.uk/Tools/msa/clustalw2/). Signal peptide analysis was performed using SignalP 4.1 (http://www.cbs.dtu.dk/services/SignalP/). NetPhos 2.0 Server (http://www.cbs.dtu.dk/services/NetPhos/) was used to predict the Phosphorylation sites. The physical and chemical parameters of DarmOrco were computed by using ProtParam tool (http://web.expasy.org/protparam/). The neighbor-joining tree of Orco orthologs from various insect species was constructed using MEGA5.0 software^57^. Topology and transmembrane domain predictions were performed using TMHMM Server v. 2.0 (http://www.cbs.dtu.dk/services/TMHMM/).

### Expression profile analysis of DarmOrco

The transcripts of different tissues, developments, and RNAi treated adults were measured by using a CFX-96 real-time PCR Detection System (Bio-Rad, Hercules, CA, USA) and the Roche SYBR Green system (Roche Diagnostics GmbH, SandhoferStraße, Mannheim, Germany). Actin gene (GenBank accession number: KJ507199.1) of *D*. *armandi* was used as endogenous control to normalize the target gene expression. The primers of the target and reference genes were designed by Primer Express 5.0 (Applied Biosystems, Carlsbad, CA) (Table 1). qRT-PCR reactions were conducted in 20 μL reaction mixtures, each containing 10 μL of 2 × SYBR Premix Ex Taq (Roche Diagnostics GmbH, Sandhofer Straße, Mannheim, Germany), 0.3 μL of each primer (10 μM), 1_ _μL of cDNA, and 8.4 μL of sterilized H_2_O. The qRT-PCR cycling conditions were as follows: 95 °C for 30 s and 40 cycles of 95 °C for 5 s, 60 °C for 20 s and 72 °C for 20 s; melt curves stages at 95 °C for 15 s; 60 °C for 1 min; and 95 °C for 15 s. Experiments for test samples, endogenous control, and negative control were performed in triplicate to ensure reproducibility. Relative quantification was performed by using the comparative 2^–ΔΔCt^ method^58^. All data were normalized to endogenous actin levels from the same tissue samples.

### RNAi

The siRNA used was commercially synthesized by Ribobio (Guangzhou, China). The target sequence used for knocking down DarmOrco was 5′-GAUGAUCUAAAGGGCGUCUTT-3′. The siRNA was dissolved in RNase-free water. Before injection, a 1% agarose plate was made and placed on an ice tray. *D*. *armandi* adults under 70% ethanol anesthesia were immobilized on the agarose plate with the abdomen directed airward using manual forceps. Afterwards, 0.05 μL DEPC treated water or siRNA solution (0.1 μM) was injected into the conjunctivum between the second and third abdominal segments of each *D*. *armandi* using a PLI-100 Pico-Injector (Harvard Apparatus, Holliston, MA, USA). Each treatment contains 40 adult beetles in triplicates. After injection, *D*. *armandi* adults were kept in a refrigerator at 4 °C. Six adults in triplicate were selected per 24_ _h (three times in total), frozen in liquid nitrogen, and then stored at −80 °C before qRT-PCR analysis (see above).

Six beetles injected for 72 h of each sex were tested for the compounds, and repeated three times each.

### EAG assay

EAGs were used to record the antennal responses of siRNA-injected, water-injected, and non-injected *D*. *armandi* to 11 major volatiles of its host. The concentration of all chemicals was 1 μg/μL in liquid paraffin. Pure liquid paraffin was used as a blank control. The antennae were carefully removed at the base, and were attached to electrode holders with electrode gel. Filter paper strips (4 × 30 mm) were loaded with 20 μL of each chemical solution and then inserted into a glass Pasteur pipette. The tip of the pipette was inserted approximately 3 mm into a small hole in the wall of a metal tube (9 mm diameter × 12 cm long) directed at the antennal preparation. An air stimulus controller (ModelCS-55, Syntech, Hilversum, The Netherlands) was used for air and odor delivery. A constant flow (300 mL/min) of activated carbon-filtered air passed over the antenna through the open end of the metal tube positioned 5 mm from the antenna. During odor stimulation, 30 mL/min of air was applied through the Pasteur pipette into the main air flow for 0.2 s. Antennae were stimulated thrice with each substance at 30 s intervals. EAG recordings were made on an IDAC-2 recording unit with amplifier and computer board (Syntech), and then stored on a hard disk drive.

### Data analysis

Data from qRT-PCR and EAG tests were analyzed using SPSS 17.0 (IBM SPSS Statistics, Chicago, IL, USA). ANOVA and Duncan’s new multiple range test (P = 0.05) were used to determine whether differences in DarmOrco mRNA levels or EAG responses were significant among different treatment groups.

## Additional Information

**How to cite this article**: Zhang, R. *et al*. Silencing of the olfactory co-receptor gene in *Dendroctonus armandi* leads to EAG response declining to major host volatiles. *Sci. Rep*. **6**, 23136; doi: 10.1038/srep23136 (2016).

## Figures and Tables

**Table 1:** Primers used in the research.

Primer use	Primer name	Primer sequence (5′-3′)
Cloning Orco fragment	DarmOrcoF	GCNATHAARTAYTGGGT
	DarmOrcoR	TTYTGRCAYTGYTGRCAYAC
3′-RACE PCR	DarmOrco-3′-GSP	CGTATGGAGTGGCTCTGTTGC
	DarmOrco-3′-NGSP	AGCAACCACTTTGGGCTACCT
5′-RACE PCR	DarmOrco-5′-GSP	CGAGTGAGTAAAGCAGGTAGCC
	DarmOrco-5′-NGSP	CAACAGAGCCACTCCATACGC
Full-length validation	f DarmOrcoF	ATGATCAACAAGTTCAAAG
	f DarmOrcoR	CTTGAGTTGCACCAGCACCAT
SYBR-Green qRT-PCR	q actin-F	GGGAGAAGATGACCCAAAT
	q actin-R	GACCAGCCAAGTCCAAACG
	qOrco-F	GGCTACCTGCTTTACTCAC
	qOrco-R	CTTCAGACCCGTCATACC
	siOrco	GAUGAUCUAAAGGGCGUCUTT
